# Sonographic Identification of an Inferior Vena Cava Tear in a Penetrating Injury

**DOI:** 10.7759/cureus.93003

**Published:** 2025-09-23

**Authors:** Máire A Bourke, Deirdre Breslin, Gillian Judge, Anna O'Leary, Gerard O'Connor

**Affiliations:** 1 Emergency Medicine, Mater Misericordiae University Hospital, Dublin, IRL

**Keywords:** emergency medicine, extended focused assessment with sonography for trauma, inferior vena cava injury, major haemorrhage, penetrating abdominal injury, trauma, ultrasound imaging

## Abstract

Injuries to the inferior vena cava (IVC) from penetrating trauma are uncommon but carry a high risk of fatality due to the potential for rapid, massive haemorrhage. We present the case of a 56-year-old man who sustained a stab wound to the right lower chest and presented in profound haemorrhagic shock. A bedside extended Focused Assessment with Sonography for Trauma (eFAST) not only identified free abdominal fluid but also provided a direct, real-time visualisation of a tear in the IVC wall with an adjacent haematoma. Despite immediate surgical intervention, the patient died from his injuries. This case highlights a rare but critical sonographic finding, suggesting that evaluating the IVC during an eFAST exam can be a valuable tool for the early detection of life-threatening vascular injuries in haemodynamically unstable patients.

## Introduction

Penetrating torso trauma is a major cause of preventable death, with uncontrolled haemorrhage responsible for nearly half of trauma fatalities [1,2]. Rapid identification of bleeding sources and timely intervention are critical, yet often difficult in unstable patients.

Focused Assessment with Sonography for Trauma (FAST) is a rapid bedside ultrasound protocol used to detect intraperitoneal and pericardial fluid. Extended FAST (eFAST) expands this to include assessment for pneumothoraces [3]. However, both protocols are limited: they identify the presence of fluid but rarely the bleeding vessel itself, and retroperitoneal injuries are easily missed because of poor sonographic access [4,5]. More recently, a modified repeated extended FAST (r-EFAST) protocol has been suggested for retroperitoneal haematomas [6], but direct sonographic evidence of caval injury remains rarely reported.

Traumatic inferior vena cava (IVC) injuries, while uncommon, carry mortality rates of 20-57%, especially in the retrohepatic segment [6-8]. Poor access for operative control, rapid blood loss, and difficulty achieving haemostasis all contribute to these outcomes. While computed tomography (CT) remains the gold standard for diagnosing retroperitoneal vascular injuries in stable patients, its use is often precluded in the unstable trauma population [9]. A bedside ultrasound may provide an opportunity to detect otherwise subtle vascular disruption in real time during the initial resuscitation phase.

This case report describes a patient with a penetrating torso injury in whom a tear of the IVC and associated haematoma were directly visualised on bedside ultrasound. To our knowledge, this represents an uncommon and underreported sonographic finding. By expanding the scope of eFAST to include targeted assessment of the IVC, we highlight a potential avenue to expedite diagnosis and initiate life-saving interventions in this highly lethal injury pattern.

## Case presentation

A 56-year-old man with no significant medical history was transported by ambulance to the emergency department after sustaining a penetrating knife wound to the right lower chest during an assault. Pre-hospital alert indicated that the patient had collapsed at the scene. Paramedics reported persistent hypotension en route despite intravenous fluid resuscitation, indicating ongoing haemorrhagic shock. No other injuries were noted.

On assessment, the patient appeared critically unwell. His airway was patent, but he had shallow, laboured respirations with a respiratory rate of 8 breaths per minute and an oxygen saturation of 86% on high-flow oxygen via a non-rebreather mask. The trachea was central. Breath sounds were markedly reduced on the right hemithorax. Circulatory assessment revealed profound shock: blood pressure was 51/23 mmHg, pulse rate was 63 beats/minute (weak and thready), and peripheral extremities were cold and mottled. His Glasgow Coma Scale (GCS) score was 7 (E2, V1, M4).

Primary survey identified a 3-cm sucking stab wound to the right lower lateral chest wall with active bleeding. Initial interventions included the application of an occlusive dressing, insertion of a right intercostal chest drain (which immediately evacuated approximately 1.2 L of blood), and activation of a massive transfusion protocol. The patient received packed red cells, fresh frozen plasma, and platelets in a 1:1:1 ratio.

**Figure 1:**
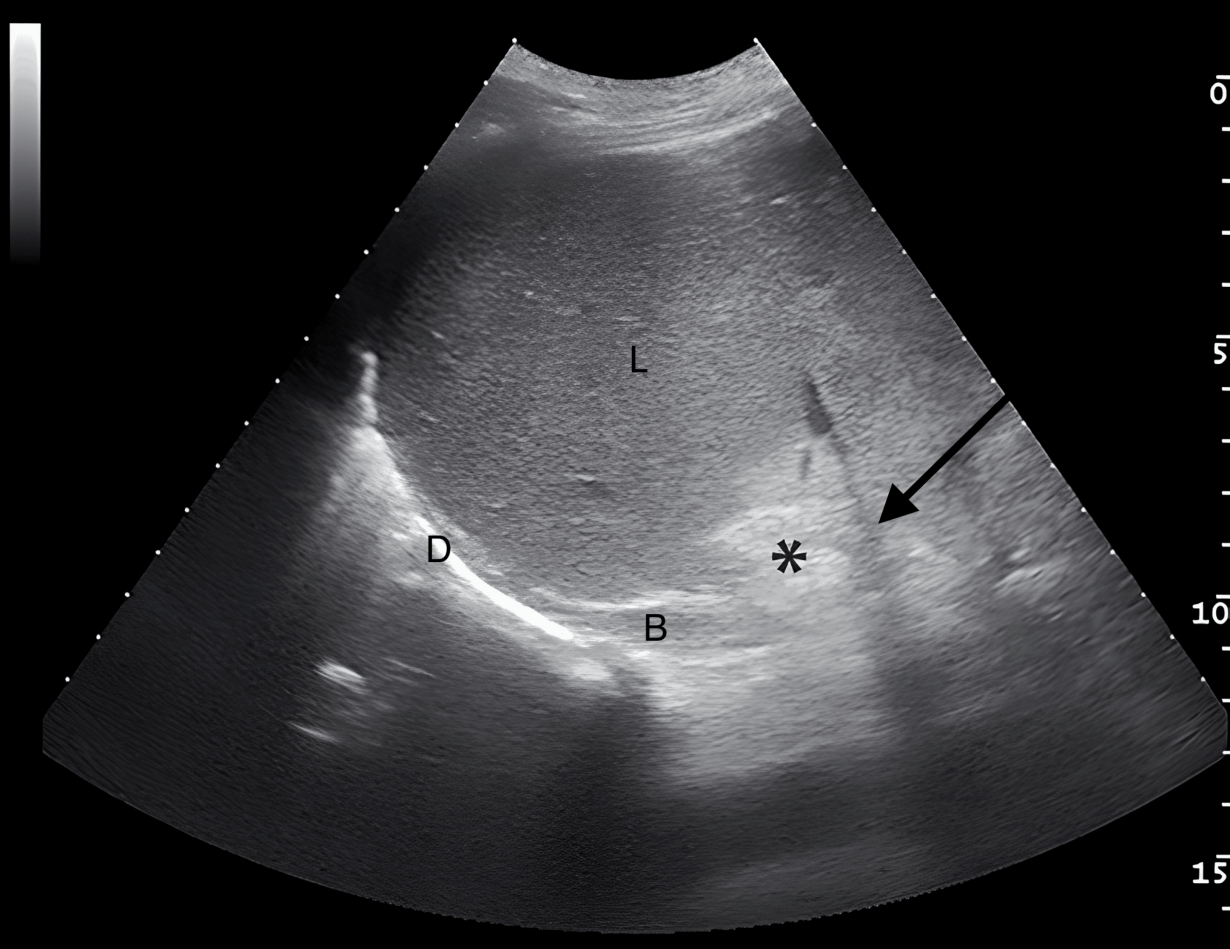
Ultrasound image of the right upper quadrant demonstrating the liver (L), inferior vena cava (arrow), and a pericaval haematoma (*). Perihepatic blood (B) is visible below the diaphragm (D)

FAST revealed free fluid in the perihepatic and perisplenic regions. In the right upper quadrant view, a discontinuity of the IVC wall with an adjacent echogenic haematoma was directly visualised (Figure 1).

Baseline laboratory investigations were consistent with profound haemorrhagic shock, demonstrating a severe metabolic acidosis with a decreased pH and base excess, a low haemoglobin level, and a markedly increased lactate (Table 1). These values reflected the severity of ongoing blood loss and guided the activation of a massive transfusion protocol.

**Table 1:** Baseline laboratory investigations This table presents the initial laboratory findings on the patient's arrival at the emergency department, demonstrating severe metabolic derangement consistent with haemorrhagic shock.

Laboratory parameter	Patient's value	Reference range
pH (venous)	7.01	7.35-7.45
Base excess	-15 mmol/L	-2 to +2 mmol/L
Haemoglobin	6.5 g/dL	13.5-17.5 g/dL
Lactate	8.9 mmol/L	0.5-2.2 mmol/L

Given the patient's haemodynamic instability, he was taken immediately to the operating theatre for exploratory thoracotomy and laparotomy. Surgical exploration confirmed a diaphragmatic defect and a laceration of the infra-diaphragmatic IVC. Despite aggressive surgical and anaesthetic interventions, including attempted direct repair, packing, and ongoing transfusion, the patient developed refractory haemorrhagic shock and died intra-operatively.

## Discussion

Traumatic injury to the IVC is an uncommon but devastating event, with reported mortality rates ranging from 20% to over 50% in large series [8-10]. Outcomes are particularly poor when the retrohepatic segment is involved, due to its deep anatomical position, its proximity to the liver and diaphragm, and the technical challenges associated with surgical exposure and repair [7-9]. Many patients with penetrating IVC trauma do not survive transport to the hospital, and those who do often deteriorate rapidly due to exsanguination despite aggressive resuscitation [8]. Survival depends on prompt recognition and expedited operative management.

Historically, IVC injuries were almost exclusively diagnosed at laparotomy. Cross-sectional imaging, while highly informative, is reserved for the minority of haemodynamically stable patients able to undergo CT [11]. For unstable patients, bedside ultrasound is often the only feasible modality. The eFAST is well established for detecting intraperitoneal or pericardial fluid [3], yet its value in identifying vascular disruption has been far less explored. Most reports describe indirect sonographic signs such as retroperitoneal haematoma or Doppler abnormalities [12,13]. To our knowledge, this case is one of the few to demonstrate the direct visualisation of a disrupted IVC wall with an associated haematoma during bedside ultrasound.

It is important to emphasise that IVC assessment is not part of the standard FAST or eFAST protocols and is more appropriately considered within the broader framework of point-of-care ultrasound (POCUS). Nonetheless, incorporating a focused evaluation of the IVC into right upper quadrant or subcostal views may yield critical information, especially when a perihepatic haematoma is identified. This approach reflects the evolution of trauma ultrasound from a tool limited to free fluid detection toward a broader application encompassing thoracic and vascular assessment [14].

From a therapeutic perspective, several strategies have been described to temporise IVC bleeding before definitive repair. These range from conventional surgical options such as IVC ligation, balloon tamponade, and atriocaval shunting to more contemporary endovascular interventions including stent placement and resuscitative endovascular balloon occlusion of the aorta (REBOA) [15-17]. Despite these innovations, retrohepatic IVC injuries continue to carry high operative mortality, highlighting the need for improved diagnostic and treatment strategies [18].

This case adds to the limited literature by showing that POCUS can detect life-threatening vascular injury in real time, extending its role beyond the identification of haemoperitoneum. Greater awareness of this application may encourage trauma clinicians to more systematically interrogate the IVC during ultrasound, particularly in patients with penetrating right upper quadrant trauma. Future studies are warranted to clarify the diagnostic yield, optimal indications, and outcome benefits of IVC assessment in trauma ultrasound.

## Conclusions

This case underscores the importance of considering major vascular injury in patients with penetrating torso trauma and haemodynamic instability. The direct sonographic visualisation of an IVC tear with associated haematoma is rare but may provide a valuable diagnostic clue to guide urgent decision-making. Further research is needed to define when systematic IVC evaluation adds value and whether it improves outcomes in penetrating torso trauma. 
